# The complete mitochondrial genome of *Iphisa elegans* (Reptilia: Squamata: Gymnophthalmidae)

**DOI:** 10.1080/23802359.2020.1797549

**Published:** 2020-07-30

**Authors:** Jean-Pierre Vacher, Sophie Manzi, Miguel Trefaut Rodrigues, Antoine Fouquet

**Affiliations:** a Laboratoire Évolution et diversité biologique (EDB), UMR5174, CNRS-UPS-IRD, Bât. 4R1, Université Paul Sabatier, Toulouse, France; b Departamento de Zoologia, Instituto de Biociências, Universidade de São Paulo, São Paulo, Brazil

**Keywords:** Reptilia, Gymnophthalmidae, Amazonia, mitochondrial genome

## Abstract

The complete mitogenome of the lizard *Iphisa elegans* Gray, 1851 was sequenced using a shotgun approach on an Illumina HiSeq 3000 platform, providing the first mitogenome for Gymnophthalmidae. The genome was 18,622 bp long, with 13 protein-coding genes, two rRNA (12S and 16S), and 22 tRNA, as well as the control region. A maximum likelihood phylogenetic analysis including *I. elegans* and all other available mitogenomes of Squamata provided a tree in accordance with previous phylogenetic relationships inferred for Squamata.

*Iphisa elegans* Gray, 1851 is a lizard of the family Gymnophthalmidae distributed throughout Amazonia. The taxonomy of *I. elegans* is not well resolved, since several mitochondrial lineages and morphotypes suggested the existence of undescribed species currently embedded in a single taxon (Nunes et al. 2012). Molecular data can significantly contribute in resolving the systematics and species boundaries within this genus but available genomic data are still scarce. Here, we describe the complete mitochondrial genome of *Iphisa elegans*.

A male of *Iphisa elegans* was collected in the Nouragues Reserve in French Guiana (N4.0716, W52.7325). Genomic DNA was isolated from liver tissue using the Wizard Genomic extraction protocol (Promega, Madison, WI). We then used 200 ng of DNA to create a DNA sequencing library at the Genotoul-GeT-PlaGe sequencing platform of Toulouse (France) with TruseqNano LT kit Illumina (Illumina Inc., San Diego, CA). The library was hybridized and sequenced on a 1/24th of lane of an Illumina HiSeq 3000 flow cell. Over 32 million paired-end read of 150 bp were obtained. The mitochondrial genome was assembled using an iterative mapping strategy (Besnard et al. 2014). We obtained a circular sequence of 18,622 bp in length. The overall base composition was as follows: A (31.3%), C (20.3%), G (18.8%), and T (29.7). We annotated the mitogenome with the MITOS webserver (Bernt et al. 2013). We validated the coding regions using Geneious version 9.0.5 (Kearse et al. 2012). The annotated sequence was deposited in GenBank (accession no. MT472615).

We then used MAFFT v.7 (Katoh and Standley 2013) to align the mitogenome of *Iphisa elegans* with all available mitochondrial genomes of Squamata retrieved from GenBank. The gene order was fully conserved in this clade, and we conducted a maximum likelihood phylogenetic analysis with RAxML v. 8.2.4 (Stamatakis 2014) excluding the control region and using *Sphenodon punctatus* to root the tree. The resulting tree (Figure 1) recovered a monophyletic Gymnophtalmidae, sister to a clade containing Chamaeleonidae, Agamidae, and Serpentes (Pyron et al. 2013; Goicoechea et al. 2016). These data, which represent the first mitogenome for the genus and the family Gymnophthalmidae, will likely serve as reference for further studies on these lizards.

**Figure 1.**
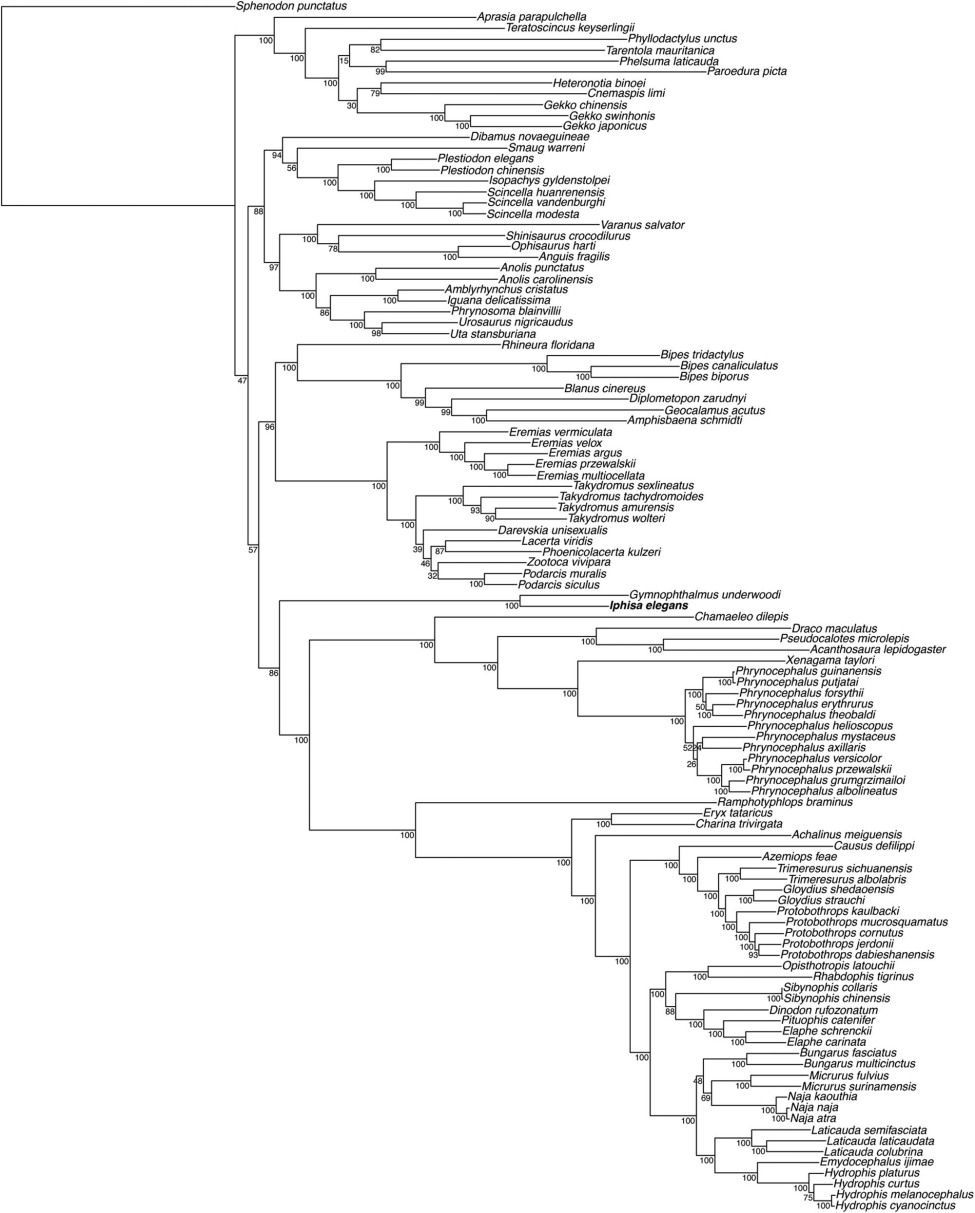
Maximum-likelihood phylogeny of Squamata inferred with a GTR + G model from all available mitochondrial genomes (excluding the control region) in this clade, and including partial mitochondrion for *Gymnophtalmus underwoodi*. We used *Sphenodon punctatus* to root the tree. The new mitogenome sequence is represented in bold. The bootstrap values (based on 100 iterations and 100 independent maximum likelihood searches) are indicated for each internal node.

## Data Availability

The data that support the findings of this study are openly available in figshare at https://doi.org/10.6084/m9.figshare.12349988.v1. The complete sequence can be accessed in GenBank at https://www.ncbi.nlm.nih.gov/nuccore/MT472615.1. The DNA sample is stored in the EDB collection (Laboratoire Évolution et Diversité Biologique, Toulouse, France) curated by Antoine Fouquet under accession number IPHFG.
